# Availability and convenience of sarcopenia screening and diagnostic tools in Chinese primary care

**DOI:** 10.3389/fpubh.2026.1859478

**Published:** 2026-06-11

**Authors:** Jinyi Zhang, Shumin Ren, Runjuan Qiao, Yiru Ma, Xiaoying Xie, Kang An, Qiaoli Su, Dan Jia

**Affiliations:** 1 West China School of Medicine, Sichuan University, Sichuan University Affiliated Chengdu Second People's Hospital, Chengdu Second People's Hospital, Chengdu, China; 2 School of Computer Science and Artificial Intelligence, Ulster University, Belfast, United Kingdom; 3 General Practice Ward/International Medical Center Ward, General Practice Medical Center, West China Hospital, Sichuan University, Chengdu, Sichuan, China; 4 Department of Public Health, Zhaotong Health Vocational College, Zhaotong, Yunnan, China; 5 Department of Internal Medicine, The Fifth People's Hospital of Sichuan Province, Chengdu, Sichuan, China; 6 Wangjiang Hospital, Sichuan University, Chengdu, China; 7 Department of Outpatient, West China Hospital, Sichuan University, Chengdu, China

**Keywords:** diagnosis, feasibility, primary care, sarcopenia, screening

## Abstract

**Background:**

Although several guidelines recommend tools for sarcopenia screening and diagnosis, their feasibility in Chinese primary health care (PHC) remains unclear. This study evaluated the availability and convenience of sarcopenia-related tools from the perspective of routine PHC practice.

**Methods:**

This two-stage expert-based evaluation was conducted between March 2023 and June 2024. In phase 1, a multidisciplinary core panel (*n* = 6) used a modified nominal group technique to define availability and convenience and to establish rating rules. In phase 2, an independent panel of PHC experts (*n* = 7) assessed 15 tools identified from major sarcopenia guidelines; ultrasonography was included as an emerging option. Inter-rater agreement was examined using Kendall’s coefficient of concordance.

**Results:**

Availability was defined as the ease with which clinicians could obtain the required equipment or tool and use it in routine practice, whereas convenience was defined as the overall practicality of the test in terms of operational difficulty, acceptability, and portability. Screening tools were consistently rated as the most feasible options for PHC. Among strength and performance assessments, the chair stand test, gait speed, the Short Physical Performance Battery, and the Timed Up and Go test were rated as highly available and highly convenient. Handgrip strength was highly convenient but only moderately available because dynamometers were not routinely stocked in many PHC institutions. For confirmatory assessment of muscle mass, ultrasonography showed the most favorable overall profile, with high availability and moderate convenience, whereas bioelectrical impedance analysis showed moderate convenience but limited availability. Inter-rater agreement was high for both convenience (Kendall’s W = 0.86, 95% CI 0.84–0.96, *p* < 0.001) and availability (Kendall’s W = 0.92, 95% CI 0.90–0.98, *p* < 0.001).

**Conclusion:**

In this exploratory expert-based assessment, Chinese PHC appeared well suited to sarcopenia case finding and first-line functional assessment, but confirmatory evaluation of muscle mass remained a major implementation bottleneck. A staged pathway may emphasize screening in PHC, selective referral, and targeted investment in feasible technologies such as bioelectrical impedance analysis and ultrasonography.

## Background

Sarcopenia was increasingly recognized as a muscle disease and was associated with falls, disability, hospitalization, and mortality (1). Recent reviews underscored its clinical and public health relevance, while prevalence estimates varied substantially across definitions and populations (2, 3). In China, the burden was particularly important because community-dwelling older adults frequently met criteria for possible sarcopenia even before formal confirmation (4).

Over the past decade, several groups proposed operational algorithms, notably EWGSOP2, ICFSR, AWGS 2019, and the 2021 Chinese expert consensus (2, 4–6). More recent international work also highlighted the lack of full harmonization across definitions and measurement strategies (7, 8). In most algorithms, screening and initial functional assessment were relatively simple, whereas confirmation of low muscle mass often required devices that were expensive, non-portable, or unavailable outside higher-level facilities (2, 4, 5, 8).

In China, primary health care (PHC) institutions were expected to provide accessible first-contact care, chronic disease management, and public health services (9–12). However, the practical match between sarcopenia guidelines and PHC infrastructure remained uncertain. Service capacity standards emphasized essential equipment and core service functions, but sarcopenia-specific resources were not uniformly embedded in routine primary care (12).

This gap mattered because general practitioners and other PHC professionals were well positioned to identify older adults at risk, deliver initial lifestyle advice, and coordinate referral when needed (13–15). Yet evidence on which tools were usable in Chinese primary care remained limited. We therefore conducted an expert-based evaluation to assess the availability and convenience of sarcopenia tools in PHC settings and to derive pragmatic implications for screening, diagnosis, and service design.

## Methods

### Study design

**Figure 1:**
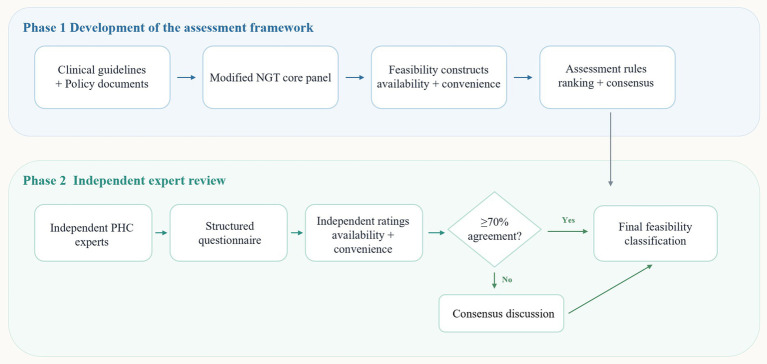
Flow diagram of the two-phase expert consensus process. PHC, primary health care. NGT, nominal group technique.

This expert-based evaluation comprised two sequential phases conducted between March 2023 and June 2024. In phase 1, a core expert panel used a modified nominal group technique (NGT) to define the feasibility constructs and the corresponding assessment rules. In phase 2, an independent expert panel applied the assessment tool to the sarcopenia instruments identified from current clinical guidelines and policy documents (Figure 1).

### Phase 1: Development of the assessment framework

NGT is a structured consensus method that supports balanced participation and transparent prioritization (14–16). The core panel included six participants: a general practitioner, a general internist, two geriatricians, a geriatric head nurse, and a methodologist. Panelists were recruited from the Chengdu Second People’s Hospital, the National Clinical Research Center for Geriatrics (West China Hospital, Sichuan University), and Sichuan Geriatric Hospital and Sichuan Geriatric Medicine Research Institute (Fifth People’s Hospital of Sichuan Province), which provided sarcopenia-related services.

Before the meeting, the research team compiled relevant clinical guidelines and PHC policy documents to support focused discussion. The panel addressed two questions: (1) how should availability and convenience of sarcopenia examinations be defined for PHC settings? and (2) how should these constructs be rated in a practical assessment tool? Participants first generated ideas independently, then discussed them in a structured round-robin format, and finally ranked candidate definitions and scoring approaches using a 5-point importance scale. The final framework was agreed by consensus after review of the ranked items.

### Phase 2: Independent expert review

The independent review panel evaluated the feasibility of sarcopenia-related tools from the perspective of routine PHC practice. Eligibility criteria included employment in a PHC institution, familiarity with sarcopenia guidelines, at least 5 years of practical experience in sarcopenia-related management, and a senior professional title. Experts were recruited through the Western China General Practice Working Group. Because only a limited number of PHC institutions in Western China were actively engaged in sarcopenia-related practice at the time of the study, the pool of eligible experts was small.

A structured online questionnaire was developed on the basis of the phase 1 framework and the guideline review. Fifteen tools were assessed: four case-finding or screening tools and eleven diagnostic or related assessment tools. Ultrasonography was included because of its growing clinical relevance. The structured online questionnaire included two parts: expert characteristics and a tool-level rating matrix. For each tool, experts reviewed a brief description, required equipment, and relevant guideline source, and then assigned one availability category and one convenience category using the definitions generated in phase 1. Experts completed the questionnaire independently. Items that did not meet the prespecified 70% agreement threshold were reviewed in a structured consensus discussion, and final classifications were assigned according to the agreed consensus procedure. Experts first completed ratings independently and were blinded to other experts’ ratings and comments; only anonymized aggregated distributions were used in the subsequent consensus discussion.

### Statistical analysis

Statistical analyses were performed using IBM SPSS Statistics for Windows, version 24.0. In Phase 1, NGT panelists anonymously rated candidate definitions and rating approaches on a 1–5 Likert scale, with higher scores indicating greater perceived importance or suitability. For each candidate item, an NGT score was calculated by summing the ratings across panelists, and items were ranked according to the total score. In Phase 2, Kendall’s coefficient of concordance (W) was used to assess overall inter-rater agreement across all 15 tools. Item-level agreement was predefined as agreement by at least five of seven experts on the same category, corresponding to a threshold of ≥70%. Given the small number of experts and the presence of tied ordinal ratings, 95% confidence intervals for Kendall’s W were estimated using expert-level bootstrap resampling, with raters resampled as the unit of analysis. A W value of 0.70 or higher was considered to indicate substantial agreement, and *p* < 0.05 was considered statistically significant.

### Ethics statement

All experts participated voluntarily and provided informed consent. The study was approved by the Ethics Committee of West China Hospital, Sichuan University (No. 2019–1,155). No patient data were collected, and all survey data were handled anonymously.

## Results

### Phase 1: Consensus on feasibility concepts

The NGT process identified two practical domains for evaluating sarcopenia-related tools in primary health care: availability and convenience. The prioritization results are shown in Supplementary Tables S1–S4. For availability, the highest-ranked definition was “the ease with which clinicians could obtain the required equipment or tool and use it in routine practice,” which received the maximum possible score (30/30 points). The highest-ranked operational approach for availability was a three-category classification (+++, ++, +), which received 22 points and ranked first, slightly ahead of a multi-item Likert-based approach (21 points). For convenience, the highest-ranked definition emphasized the overall practicality of the test, including operational difficulty, acceptability, and portability (29 points). The highest-ranked assessment approach for convenience was a simple three-level overall rating (***, **, *), which received 26 points, compared with 24 points for separate Likert ratings of each component and 21 points for a summed Likert-based stratification. The panel adopted a simple three-level overall rating system for both constructs.

Availability was therefore defined as the ease with which clinicians could obtain the required equipment or tool and use it in routine practice, and was categorized as high availability (+++; equipment already available), moderate availability (++; equipment not routinely available but relatively easy to obtain), or low availability (+; equipment not available and not easy to obtain). Convenience was defined as the overall practicality of the test, incorporating operational difficulty, acceptability, and portability, and was categorized as high (***), moderate (**), or low (*).

### Phase 2: Expert panel characteristics and agreement

The independent review was completed between May and June 2024. Seven GPs responsible for sarcopenia management were recruited from seven PHC institutions. Inter-rater agreement was high for both convenience and availability ratings. For convenience, Kendall’s coefficient of concordance was 0.86 (95% CI, 0.84–0.96; χ^2^ = 84.26, df = 14, *p* < 0.001). For availability, Kendall’s coefficient of concordance was 0.92 (95% CI, 0.90–0.98; χ^2^ = 90.13, df = 14, *p* < 0.001). Items with lower item-level agreement were discussed further before final classification. For convenience, BIA showed the lowest agreement, with 4 of 7 experts (57.1%) rating it as moderately convenient and 3 of 7 (42.9%) rating it as highly convenient. After discussion, BIA was classified as having moderate convenience overall. For availability, ultrasonography showed the lowest item-level agreement, with ratings split between high availability (4/7, 57.1%) and moderate availability (3/7, 42.9%). Following structured discussion, ultrasonography was classified as having high availability overall.

### Feasibility of screening and functional assessment tools

Table 1 summarizes the ratings for screening tools and the main strength and performance assessments recommended in current guidelines. Screening instruments were consistently judged to be the most feasible options in PHC. SARC-F, SARC-CalF, and calf circumference were rated as highly available and highly convenient because they required only a questionnaire and/or a simple tape measurement. The Ishii screening tool was also considered highly convenient, but its availability was slightly lower because it depends on grip-strength measurement.

**Table 1:** Feasibility of screening and functional assessment tools for sarcopenia in Chinese primary care.

Use	Domain	Tool	Brief description	Equipment required	Guideline(s)	Availability	Convenience
Screening	Case finding	SARC-F	Self-report on strength, walking assistance, chair rise, stairs, and falls.	Questionnaire	EWGSOP2; AWGS 2019; ICFSR 2018	+++	***
Screening	Case finding	SARC-CalF	SARC-F combined with calf circumference.	Questionnaire; inelastic tape	AWGS 2019; CGS 2021	+++	***
Screening	Case finding	Ishii tool	Equation using age, grip strength, and calf circumference.	Dynamometer; inelastic tape	EWGSOP2	++	***
Screening	Case finding	Calf circumference	Calf measured at the widest point.	Finger-ring test or inelastic tape	AWGS 2019; EWGSOP2; CGS 2021; ICFSR 2018	+++	***
Diagnosis	Muscle strength	Handgrip strength	Dynamometer-based muscle strength test.	Spring or hydraulic dynamometer	EWGSOP2; AWGS 2019; ICFSR 2018; CGS 2021	++	***
Diagnosis	Muscle strength	Chair stand test	Time required to rise five times from a chair.	Chair; stopwatch	AWGS 2019; EWGSOP2; CGS 2021	+++	***
Diagnosis	Performance	Gait speed	Usual walking speed over 4 m or 6 m.	Walkway; marker; stopwatch	EWGSOP2; AWGS 2019; ICFSR 2018; CGS 2021	+++	***
Diagnosis	Performance	SPPB	Composite of balance, gait speed, and chair stand tests.	Walkway; chair; marker; stopwatch	AWGS 2019; EWGSOP2; CGS 2021	+++	***
Diagnosis	Performance	TUG	Stand up, walk 3 m, turn, return, and sit.	Chair; 3-m walkway; marker	EWGSOP2	+++	***
Diagnosis	Performance	400-m walk	Twenty 20-m laps completed as quickly as possible.	20-m corridor; marker; stopwatch	EWGSOP2	+++	***

AWGS, Asian Working Group for Sarcopenia; CGS, Chinese Geriatrics Society; EWGSOP, European Working Group on Sarcopenia in Older People; ICFSR, International Clinical Practice Guidelines for Sarcopenia; SPPB, Short Physical Performance Battery; TUG, Timed Up and Go. Availability was classified as high (+++), moderate (++), or low (+). Convenience was classified as high (***), moderate (**), or low (*).

Among strength assessments, handgrip strength was rated as highly convenient but only moderately available because dynamometers were not routinely stocked in many PHC institutions. By contrast, the chair stand test was rated as highly available and highly convenient because it required only a chair and a timer. Among physical performance measures, gait speed, the Short Physical Performance Battery (SPPB), and the Timed Up and Go (TUG) test were all considered highly feasible. The 400-m walk test was classified as highly feasible overall, although some experts considered it less practical because of time and space requirements.

### Feasibility of muscle mass confirmation

Table 2 summarizes the confirmatory tools for muscle quantity. Ultrasonography showed a favorable overall profile among the equipment-dependent methods, with high availability but only moderate convenience. BIA was classified as moderately convenient, but its availability remained low for routine PHC use. DXA, CT, and MRI were all considered less practical for routine PHC use because of infrastructure and workflow requirements.

**Table 2:** Feasibility of confirmatory muscle-mass assessment tools for sarcopenia in Chinese primary care.

Use	Domain	Tool	Brief description	Equipment required	Guideline(s)	Availability	Convenience
Diagnosis	Muscle quantity	DXA	Estimates appendicular skeletal muscle mass.	DXA device	EWGSOP2; AWGS 2019; ICFSR 2018; CGS 2021	+	*
Diagnosis	Muscle quantity	BIA	Estimates total or appendicular skeletal muscle mass.	BIA device	AWGS 2019; EWGSOP2; CGS 2021	+	**
Diagnosis	Muscle quantity	Ultrasound	Measures muscle thickness and cross-sectional area.	Ultrasound device or portable ultrasound	Emerging option	+++	**
Diagnosis	Muscle quantity	CT	Measures lumbar or thigh muscle cross-sectional area.	Computed tomography scanner	EWGSOP2	+	*
Diagnosis	Muscle quantity	MRI	Measures lumbar or thigh muscle cross-sectional area.	Magnetic resonance imaging scanner	EWGSOP2	+	*

BIA, bioelectrical impedance analysis; CT, computed tomography; DXA, dual-energy X-ray absorptiometry; MRI, magnetic resonance imaging. Availability was classified as high (+++), moderate (++), or low (+). Convenience was classified as high (***), moderate (**), or low (*).

## Discussion

This study provided a pragmatic PHC-oriented appraisal of sarcopenia tools in China. Primary care was well suited to case finding and first-line functional assessment, but full diagnostic confirmation of low muscle mass remained constrained by equipment access. That distinction was clinically important because sarcopenia algorithms increasingly relied on staged assessment rather than universal imaging or body-composition testing (2, 4–8, 14, 16).

The most immediately implementable tools were the simplest ones. Self-report instruments and calf circumference could be deployed quickly, required minimal training, and fit routine consultations. This supported opportunistic case finding in primary care, especially for older adults with frailty, falls, multimorbidity, or functional decline (17). Our findings were aligned with previous work showing that sarcopenia management in everyday practice depended heavily on simple, portable tools and clear referral pathways (13, 14, 16).

For strength and performance assessment, the distinction between technical validity and practical deployability mattered (18). Handgrip strength was widely endorsed and clinically informative, but the required dynamometer was still not standard equipment in many Chinese PHC institutions. In contrast, the chair stand test and gait speed could be implemented immediately with minimal cost. From a workflow perspective, these tests may have offered the best balance between validity and feasibility when PHC teams needed rapid assessment within ordinary consultations or community visits (13, 14). SPPB and TUG were also considered practical, although their use may be more sensitive to time constraints in busy clinics (19).

Assessment of muscle mass was the main bottleneck. Expert ratings favored ultrasound overall, while BIA offered a strong convenience profile despite limited current availability. This was consistent with current debates in the field: BIA was relatively affordable and portable but could be influenced by hydration and device-specific algorithms, whereas ultrasound was attractive for bedside use but still faced challenges related to operator training, anatomical measurement sites, and cut-off standardization (20). For PHC systems that wanted to expand sarcopenia services, BIA may have been the most realistic first investment, with ultrasound representing a promising option where staff training and quality assurance could be supported (21).

These findings also had policy implications. Chinese PHC has a central role in healthy ageing, chronic disease follow-up, and community-based prevention, yet sarcopenia was not always operationalized as a routine component of PHC service packages (9). A practical strategy would be to embed sarcopenia within existing geriatric assessment and falls-prevention workflows, beginning with case finding and simple functional tests, followed by referral or on-site confirmation where feasible (22, 23). Recent Asian and Chinese studies similarly supported staged community strategies rather than immediate reliance on high-resource confirmatory testing (24, 25). At the same time, implementation should avoid indiscriminate labeling or unnecessary testing; screening must remain linked to actionable interventions and clinically meaningful pathways (26).

**Figure 2:**
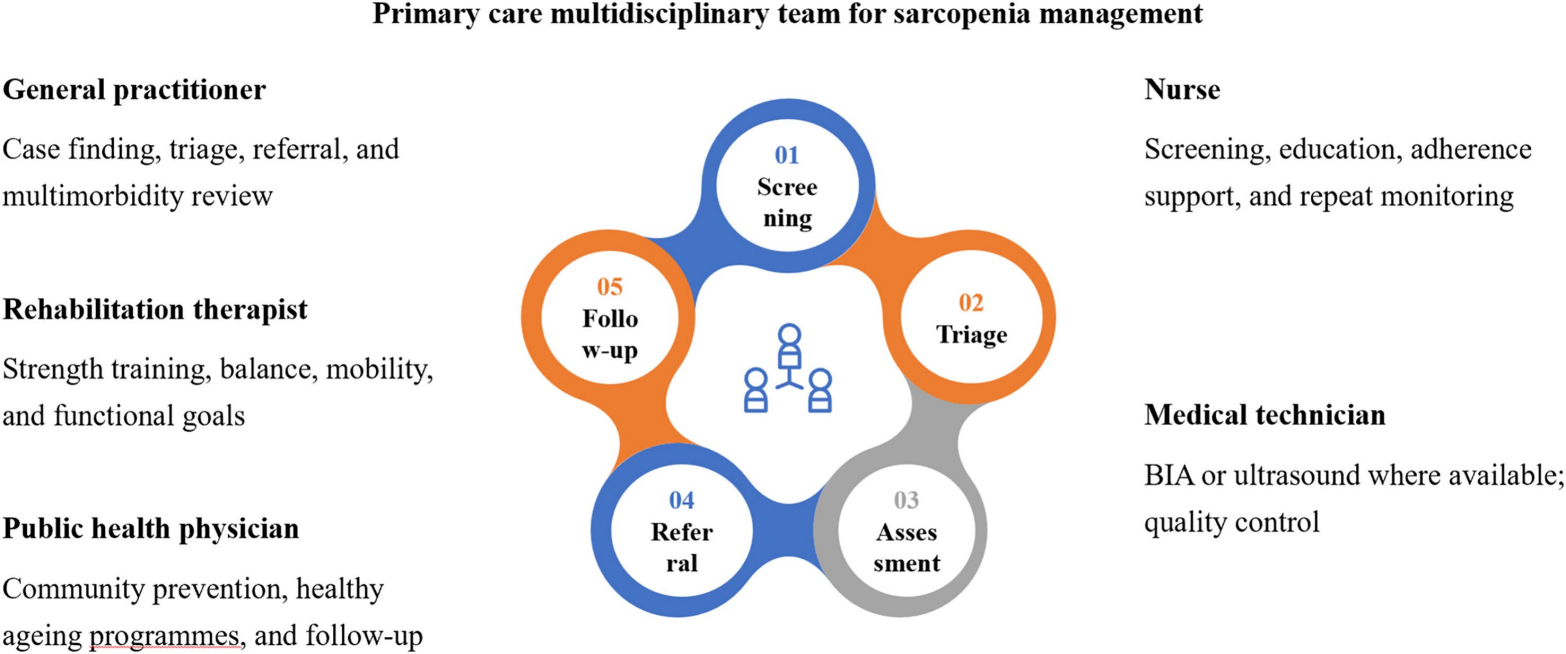
Multidisciplinary primary care model for sarcopenia.

The multidisciplinary model shown in Figure 2 illustrated how PHC teams could operationalize this approach. General practitioners could lead case finding and care coordination; nurses could support screening and follow-up; rehabilitation therapists could guide resistance exercise and functional training; medical technicians could assist with BIA or ultrasound where available; and public health physicians could integrate sarcopenia prevention into community programmes for healthy ageing (27, 28).

### Strengths and limitations

This study has several strengths. It used a two-stage design, drew on a multidisciplinary core panel, and incorporated perspectives from PHC settings. The resulting framework is simple enough to be readily understood by clinicians and policymakers, yet sufficiently specific to inform implementation decisions. Several limitations should also be acknowledged. First, the expert sample was small and geographically limited to one western Chinese region, mainly because only a limited number of PHC institutions in Western China were actively engaged in sarcopenia-related practice at the time of the study. Therefore, the findings may not be statistically representative of all Chinese PHC institutions, particularly those in rural or under-resourced settings. Second, experts were recruited from institutions already engaged in sarcopenia-related care, which may have led to more favorable ratings of equipment-dependent tools such as ultrasound and BIA. Third, the study assessed perceived availability and convenience rather than direct workflow data; it did not measure diagnostic accuracy, consultation time, cost, training burden, cost-effectiveness, referral completion, patient outcomes, or implementation fidelity. Fourth, although a qualitative sensitivity check was added, the analysis remained descriptive and no external validation study, confidence intervals, subgroup analyses, or formal threshold-based sensitivity analyses were performed. Finally, patients, caregivers, and members of the public were not involved, so patient usability, acceptability, willingness to undergo testing, and equity concerns require further study.

## Conclusion

In this exploratory, expert-based assessment, Chinese primary care appeared well positioned to identify older adults at risk of sarcopenia and to undertake first-line functional assessment using simple, low-cost tools. The main implementation barrier was the confirmation of low muscle mass. A staged pathway that prioritizes screening, chair stand and gait speed assessment, selective referral, and targeted investment in BIA or ultrasound may be feasible in selected settings, but these recommendations should be tested before broad implementation. Future multicentre studies should measure diagnostic accuracy, workflow efficiency, training requirements, costs, patient acceptability, and patient outcomes in real PHC settings.

## Data Availability

The raw data supporting the conclusions of this article will be made available by the authors, without undue reservation.
