# Supplementary material for: Availability and convenience of sarcopenia screening and diagnostic tools in Chinese primary care
Source: Front Public Health. 2026 Jun 11;14:1859478. doi: 10.3389/fpubh.2026.1859478 (PMC13293883; doi:10.3389/fpubh.2026.1859478)
Supplement: Supplementary file 1 [file Table_1.DOCX]

**Additional File 1**

**Prioritization tables from the modified nominal group technique sessions**

This supplementary file presents the ranked ideas generated during the modified nominal group technique process. Scores reflect the importance ratings assigned by the six panelists during the consensus exercise.

Supplementary Table S1. Top-ranked definition of availability in the modified NGT session

| **Idea** | **Respondent 1** | **Respondent 2** | **Respondent 3** | **Respondent 4** | **Respondent 5** | **Respondent 6** | **Points** | **Ranking** |
| --- | --- | --- | --- | --- | --- | --- | --- | --- |
| Definition A | 5 | 5 | 5 | 5 | 5 | 5 | 30 | 1st |

Notes. Definition A: the ease with which clinicians can access the required equipment or tool and use it in practice.

Supplementary Table S2. Top-ranked approaches to rating availability in the modified NGT session

| **Idea** | **Respondent 1** | **Respondent 2** | **Respondent 3** | **Respondent 4** | **Respondent 5** | **Respondent 6** | **Points** | **Ranking** |
| --- | --- | --- | --- | --- | --- | --- | --- | --- |
| Assessment A | 5 | 3 | 4 | 4 | 4 | 2 | 22 | 1st |
| Assessment B | 3 | 4 | 3 | 3 | 3 | 5 | 21 | 2nd |

Notes. Assessment A: three-category classification using existing equipment (+++), not routinely available but easy to obtain (++), and not available and not easy to obtain (+). Assessment B: Likert-type rating scale.

Supplementary Table S3. Top-ranked definition of convenience in the modified NGT session

| **Idea** | **Respondent 1** | **Respondent 2** | **Respondent 3** | **Respondent 4** | **Respondent 5** | **Respondent 6** | **Points** | **Ranking** |
| --- | --- | --- | --- | --- | --- | --- | --- | --- |
| Definition 1 | 5 | 3 | 5 | 3 | 5 | 3 | 24 | 2nd |
| Definition 2 | 4 | 5 | 5 | 5 | 5 | 5 | 29 | 1st |

Notes. Definition 1: degree of portability of sarcopenia tests. Definition 2: overall practicality based on operational difficulty, acceptability, and portability.

Supplementary Table S4. Top-ranked approaches to rating convenience in the modified NGT session

| **Idea** | **Respondent 1** | **Respondent 2** | **Respondent 3** | **Respondent 4** | **Respondent 5** | **Respondent 6** | **Points** | **Ranking** |
| --- | --- | --- | --- | --- | --- | --- | --- | --- |
| Assessment 1 | 5 | 3 | 4 | 3 | 3 | 3 | 21 | 3rd |
| Assessment 2 | 4 | 5 | 4 | 4 | 4 | 5 | 26 | 1st |
| Assessment 3 | 4 | 4 | 5 | 4 | 4 | 3 | 24 | 2nd |

Notes. Assessment 1: operational difficulty, acceptability, and portability were scored separately and then stratified. Assessment 2: overall three-level rating with high (***), moderate (**), and low (*). Assessment 3: operational difficulty, acceptability, and portability were rated separately with Likert scales.
